# Identification of potential inhibitors of omicron variant of SARS-Cov-2 RBD based virtual screening, MD simulation, and DFT

**DOI:** 10.3389/fchem.2022.1063374

**Published:** 2022-12-08

**Authors:** Xudong Lü, Cuiyue Feng, Ruijie Lü, Xiyu Wei, Shuai Fan, Maocai Yan, Xiandui Zhu, Zhifei Zhang, Zhaoyong Yang

**Affiliations:** ^1^ Institute of Medicinal Biotechnology, Chinese Academy of Medical Sciences & Peking Union Medical College, Beijing, China; ^2^ School of Pharmacy, North China University of Science and Technology, Tangshan, China; ^3^ School of Pharmacy, Jining Medical University, Rizhao, China; ^4^ North China University of Science and Technology, Tangshan, China

**Keywords:** SARS-CoV-2 omicron variant, virtual screening, drug discovery, DFT, MD simulation

## Abstract

Emergence of the SARS-CoV-2 Omicron variant of concern (VOC; B.1.1.529) resulted in a new peak of the COVID-19 pandemic, which called for development of effective therapeutics against the Omicron VOC. The receptor binding domain (RBD) of the spike protein, which is responsible for recognition and binding of the human ACE2 receptor protein, is a potential drug target. Mutations in receptor binding domain of the S-protein have been postulated to enhance the binding strength of the Omicron VOC to host proteins. In this study, bioinformatic analyses were performed to screen for potential therapeutic compounds targeting the omicron VOC. A total of 92,699 compounds were screened from different libraries based on receptor binding domain of the S-protein *via* docking and binding free energy analysis, yielding the top 5 best hits. Dynamic simulation trajectory analysis and binding free energy decomposition were used to determine the inhibitory mechanism of candidate molecules by focusing on their interactions with recognized residues on receptor binding domain. The ADMET prediction and DFT calculations were conducted to determine the pharmacokinetic parameters and precise chemical properties of the identified molecules. The molecular properties of the identified molecules and their ability to interfere with recognition of the human ACE2 receptors by receptor binding domain suggest that they are potential therapeutic agents for SARS-CoV-2 Omicron VOC.

## 1 Introduction

More than 310 million people have been infected while 5.5 million have died as a result of the COVID-19 pandemic caused by the SARS-CoV-2 virus, which affects the lower respiratory tract (Uddin et al., 2020). The management of SARS-CoV-2 pandemic has been complicated by emergence of several major SARS-CoV-2 variants. The recently identified Omicron variant of concern (VOC) has raised serious concerns with regards to the efficacies of vaccines and neutralization antibodies (Ismail, 2022).

Cases with severe forms of Omicron VOC infections presented with viral pneumonia symptoms that were similar to those of previous SARS-CoV-2 infections, including cough, fever, dyspnea, and bilateral pulmonary infiltrates (Kotfis et al., 2020). The SARS-CoV-2 is a new β-coronavirus belonging to the family of enveloped, positive-sense, single-stranded RNA viruses, approximately 1,250 nm in diameter, similar to SARS-CoV-1 and MERS-CoV (Zhu et al., 2020). The SARS-CoV-2 virus exhibits rapid human-to-human transmission, and its early atypical symptoms are difficult to manage (Wang et al., 2020). Since the start of the SARS-COV-2 pandemic, new variants have emerged, such as the Alpha (B.1.1.7), Beta (B.1.351), Gamma (P.1), Delta (B.1.617.2), and Omicron (B.1.1.529) which are associated with enhanced transmissibility and increased virulence (Xiong et al., 2022). The Delta (B.1.617.2) was first detected in India (Oct 2020) and was associated with about 97% increased transmissibility, while the Alpha (B.1.1.7), Beta, and Gamma variants are associated with 25%; 25%, and 38% transmissibility rates, respectively (Aleem et al., 2022; Singhal, 2022). The Omicron variant, which is able to evade natural and vaccine-induced immunity, has spread globally, replacing the Delta variant as the most infective variant (Singhal, 2022). Therefore, development of effective treatment strategies for infections of the Omicron Variant is urgent.

The SARS-CoV-2 genome is about 29.9 kb in size (Lu et al., 2020), encoding 7096 long polyproteins that contain four structural proteins (S,E,M,N), five accessory proteins (ORF3, ORF4a, ORF4b, ORF5, ORF8) and sixteen non-structural proteins (NSP1-16), with different functions that cooperatively enable the rapid spread and proliferation of the virus (Totura and Bavari, 2019). Among them, the spike glycoprotein (S) mediates the entry of coronaviruses into host cells (Sixto-López et al., 2021). During the infection process, the transmembrane spike glycoprotein forms prominent homotrimers on viral surfaces (Lu et al., 2020). The S1 subunit of S protein is involved in the attachment of virions to host cell membranes by interacting with the human receptor angiotensin-converting enzyme 2 (ACE2) (Hoffmann et al., 2020; Mousavizadeh and Ghasemi, 2021). We focused on receptor binding domain (RBD), which is located in the S1 subunit. Physiologically, RBD is composed of about 200 amino acid residues (residues 333–530) comprising the core and external subdomains. The outer subdomain contains disulfide-stabilized flexible loops (Han et al., 2022). The outer subdomain of RBD plays an important role in recognizing the ACE2 receptor. Therefore, RBD is an attractive target for anti-SARS-COV-2 drugs. Recent SARS-COV-2 VOCs, including the Omicron VOC, harbor multiple mutations on the RBD (Aleem et al., 2022). The Omicron VOC has fifteen mutations (Y505H, N501Y, Q498R, G496S, Q493R, E484A, T478K, S477N, G446S, N440K, K417N, S375F, S373P, S371L, and G339D) on the RBD, the highest number of mutations in a SARS-COV-2 VOC. The Y505H, N501Y, Q498R, G496S, Q493R, and S477N mutations are unique to the Omicron VOC (Han et al., 2022). These new interactions synergistically reinforce the binding of RBD and ACE2 receptors, firmly attaching the virus to the host cell membrane, enhancing its infection and spread. Among Omicron variants, RBD is the domain with the largest number of mutation sites. Identifying inhibitors of the PPI interface region on RBD can greatly inhibit viral spread. Therefore, RBD is a potential ideal therapeutic target for Omicron VOC.

Since the outbreak of COVID-19, there have been advances in structural and enzymatic studies of proteins from various components of SARS-CoV-2, which has facilitated the use of computer-aided strategies to identify viral inhibitors. Lau et al. (2021) used molecular docking, molecular dynamics simulations, and machine learning to identify candidate compounds that might inhibit the virus. Through high-throughput virtual screening and biochemical analysis, Clyde et al. (2022) identified a novel small-molecule inhibitor (MCULE-5948770040). Then, they used molecular dynamics simulations and machine learning to explain the mechanisms underlying its inhibition of the main proteases. A plant-derived natural compound has low toxicity, ease of extraction, acceptance, and a shorter trial period (Patel et al., 2022a). Patel et al. (2021a); Patel et al. (2022a) used molecular docking and molecular dynamics simulations to identify eight potential plant-derived RBD protein fusion inhibitors in two separate studies. Following binding free energy calculations and ADMET predictions, these phytochemicals are shown to be potential S protein blockers. Furthermore, plant-derived compounds have shown potential resistance for COVID-19 in virtual screening studies targeting HE glycoprotein receptor and main protease receptors (Patel et al., 2021b; Verma et al., 2021; Patel et al., 2022b). These studies identify potential small molecule inhibitors of SARS-Cov-2, providing more effective support for experimental and clinical trials.

## 2 Methods

### 2.1 Target protein structure and ligand preparation

In this study, we focused on RBD of Omicron VOC. The crystal structure was retrieved from the PDB library (PDB ID: 7WBP) (Patel et al., 2021b). All inorganic ions and water molecules were removed. The combined library (containing 92,699 compounds in total) was obtained from Approved Drugs in Major Juridications (https://zinc.docking.org/substances/subsets/world/), Enamine Coronavirus Library (https://enamine.net/), Asinex small molecule PPI inhibitors (http://www.asinex.com/), traditional Chinese medicine natural products (http://tcm.cmu.edu.tw/) and Alinda natural products (https://www.alinda.ru) databases. The corresponding structures of these compounds were downloaded from the ZINC database (http://zinc.docking.org/). The Openbabel software was used to separate the sdf files of individual small molecules.

### 2.2 Molecular docking-based virtual screening

The compounds were processed to protonation states and pdbqt file using the MGLTools software for docking. Docking was conducted *via* AutoDock Vina 1.2.3 (Trott et al., 2010; Eberhardt et al., 2021), with different parameters. The resulting docked structures were ranked according to their predicted binding energies. For the initial screening, “exhaustiveness” was set to 50. The compounds representing cluster centroids were used with a docking box of 26.25 × 45.50 × 22.50 Å and center at [−35.72, 32.42, 0.22]. The top 1,000 hits from all docking procedures were selected for further screening. For the second screening, “exhaustiveness” was set to 400. Finally, the top 10 of the 1,000 highest binding energies and best-docked conformation were considered for the next MD simulation.

In order to validate the docking procedure, we have run an enrichment test. We selected three small molecules as control molecules, lifitegrast sodium, evans blue and lumacaftor, that have been shown experimentally to have detectable binding to RBD proteins and block the recognition of RBD and ACE2 (Day et al., 2021). The wild-type RBD protein was obtained from the PDB (PDB ID: 6M0J). Ten thousand small molecules were randomly selected, and the same docking parameters as virtual screening were used for docking test.

### 2.3 Molecular dynamic (MD) simulation

The MD Simulations were performed using the Gromacs 2019.6 package (Van Der Spoel et al., 2005). Parameterization of the protein was conducted using the AMBER14SB force field (Maier et al., 2015). Then, the ligand was parametrized with the general AMBER force field (Wang et al., 2004) obtained from the AmberTools21 program (Case et al., 2021). The binding conformation with the highest affinity in docking was used as the simulated initial complex conformation. The resultant system was solvated in a dodecahedron box with TIP3P water (Jorgensen et al., 1983) molecules extending 10 Å from any atoms of the protein in any directions. Three sodium ions were added to maintain the neutrality of the system. The final system had 28,608 atoms. All bond lengths involving hydrogen atoms were constrained using the SHAKE algorithm (Ryckaert et al., 1977). The time step for integration of equations of motion was 2 fs. The particle mesh Ewald method (Darden et al., 1993) with a cutoff distance of 10 Å was used to calculate Coulomb interactions. The steepest descent method (Hratchian et al., 2010) was performed to minimize the system with a tolerance value of 1,000.0 kJ/mol/nm. Then, all systems were sequentially minimized and equilibrated in NVT and NPT ensembles. Then, the system was heated to 300 K using the v-rescale temperature coupling scheme (Bussi et al., 2007) with the NVT ensemble in 1,000 ps, followed by another 1000-ps NPT simulation *via* the Parrinello Rahman pressure coupling scheme (Martyna et al., 1994). Each of the ten systems was performed for MD simulation of 100 ns.

### 2.4 Binding free energy calculations

We further assessed the binding free energies between RBD and ligands with the MD results. The MMPBSA (Molecular Mechanics Poisson-Boltzmann Surface Area) method (Homeyer and Gohlke, 2012), implemented in the gmx_MMPBSA program (Valdés-Tresanco et al., 2021) was adopted along with AmberTools21 (Case et al., 2021). The PB model estimates only the polar component of the solvation. The non-polar component is usually assumed to be proportional to the molecule’s total solvent accessible surface area, with proportionality constant derived from experimental solvation energies of small non-polar molecules. This method has been proven to balance accuracy and computational efficiencies, especially when dealing with large systems. In the 100-ns simulation, the most stable 40-ns trajectory was selected for free energy calculation. Based on the calculated results, the 5 compounds with strongest binding abilities to RBD were used for energy decomposition, MD trajectory analysis, quantum chemical calculations and ADMET prediction.

### 2.5 Quantum chemical calculations

To establish the more precise molecular properties of the top 5 compounds, the DFT method was used to perform quantum chemical calculations with the Gaussian 16 program (Frisch et al., 2016). Geometries were optimized without any constraint with the B3LYP method (Becke, 1993; Stephens et al., 1994) using 6-311G (d,p) basis (Ditchfield et al., 1970; Hehre et al., 1972; Hariharan and Pople, 1973) with DFT-D3 empirical dispersion corrections (BJ damping) (Grimme et al., 2011). The polarizable continuum model implicit solvent model (Di Remigio et al., 2016) was used to study the effects of water solvents. The Multiwfn 3.8 program (Lu and Chen, 2012a) was used for Electrostatic potential (ESP) maps generation with the five selected molecules. Then, the electrostatic potential involved in analyses was evaluated by Multiwfn based on the highly effective proposed algorithm (Lu and Chen, 2012b; Zhang and Lu, 2021). The Visual Molecular Dynamics program (Humphrey et al., 1996) was used for visualization of ESP surface. Based on Density Functional Reactivity Theory (LIU, 2016), the lowest unoccupied molecular orbital energy (ELUMO), the highest occupied molecular orbital energy (EHOMO), and other molecular chemical descriptors such as chemical hardness (η), chemical softness (S), band gap (GAPE), electron affinity (EA), ionization potential (IP), electrophilicity index (ω), electronic potential (μ), and electronegativity (χ) were calculated.

### 2.6 Absorption, distribution, metabolism, excretion and toxicity prediction

Pharmacokinetics and toxicity are important considerations in drug development. Evaluating the absorption, distribution, metabolism, excretion and toxicity (ADMET) properties (Ferreira and Andricopulo, 2019) of compounds can help in determining whether they are a potential drug candidates. We predicted the ADMET properties of the top 5 compounds obtained from the above screening tests. The ADMET prediction was performed using the ADMETlab 2.0 online server (Xiong et al., 2021) (https://admetmesh.scbdd.com/). Human intestinal absorption (HIA), through the blood−brain barrier permeability, cytochrome P450 enzyme inhibition, hepatotoxicity, as well as mitochondrial toxicity and other important indicators were selected as predictors of ADMET.

## 3 Results and discussion

### 3.1 Virtual screening of compound Libraries against SARS-CoV-2 protein

**FIGURE 1:**
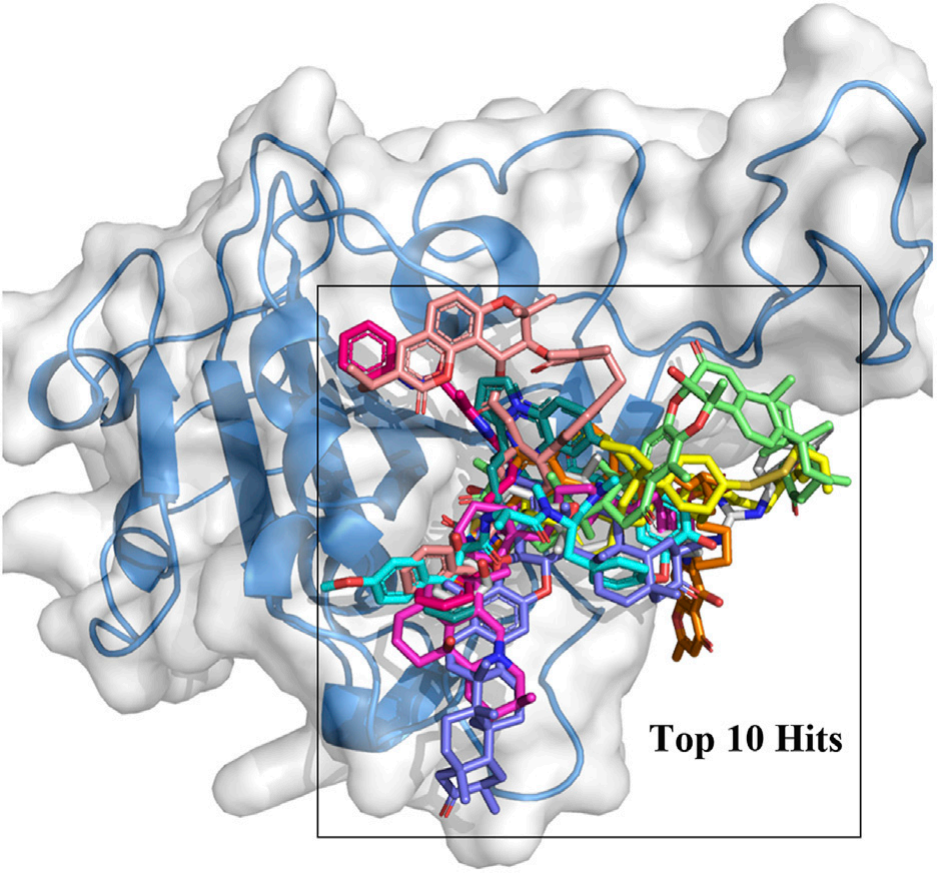
Top 10 compounds shown in different colors docked to the RBD PPI interface region.

In this study, the amino acid residues where RBD interacts with ACE2 at the PPI interface are referred to as recognition residues. The mutation sites unique to Omicron VOC are described as hotspot residues. In Supplementary Figure S1, the binding energy values of 244 compounds ranged from −8.5 to −10.7 kcal/mol in the second screening. Based on their structural diversity, 50 of these compounds were selected. Then, the binding conformation of these compounds were visually inspected (Supplementary Table S1). Candidates that had good interactions with SARS CoV-2 Omicron RBD protein were selected. After screening twice with different precisions, 10 compounds were selected for subsequent analyses (Figure 1). The docking results are shown in Table 1. Each molecule has varying degrees of contact with hotspot residues (1–4). The number of recognition residues that interacted with the 10 candidate molecules ranged from 1 to 9. The greater the number of contact recognition residues in the candidate molecule, the stronger the PPI inhibition. The top 5 candidate molecules with the highest binding affinities interacted with an average of 8 recognition residues. The top 5 molecules with the highest binding affinities showed high inhibitory activities. This strongly inhibits the recognition of ACE2 receptors by RBD. The results of the enrichment test showed that the binding energy ranking of the three control molecules was all within 1%. This result confirms the reliability of the docking procedure in this study, indicating that it can be used for candidate compound enrichment. Furthermore, we docked three control molecules with Omicron RBD protein, and the results showed that their binding affinity to proteins was weaker than that of the first 10 compounds screened. This also implies that the top 10 compounds are potential RBD protein binders.

**TABLE 1:** Docking results and binding residues of top10 compounds.

Title	Docking score	Interacting residues	Hotspot residues	Recognition residues
ZINC95919448	−10.7	TYR501 THR500 ARG498 SER496 TYR495 SER494 ARG403 HIS505 TYR453 TYR449 VAL445	4	8
ZINC85531210	−10	ARG403 ASP405 GLU406 ASN417 PHE456 LEU455 TYR453 LYS493 SER494 TYR495 SER496 HIS505 GLY502 TYR501	4	9
ZINC95610651	−9.9	TYR501 GLY502 HIS505 ARG403 SER496 TYR495 SER494 LYS493 TYR453 LEU455 TYR449	4	9
ZINC000035399302	−9.8	ARG403 HIS505 TYR453 LEU455 ASN417 SER496 TYR495 TYR501	3	5
ZINC95910594	−9.7	HIS505 ARG403 SER496 TYR495 SER494 LYS493 TYR453 PHE456 PHE490 TYR489 ALA484 TYR501	4	8
ZINC85542617	−9.6	HIS505 GLY502 TYR501 ARG498 PHE497 SER496 TYR495 SER494 LYS493	4	6
ZINC85593889	−9.6	TYR449 LEU452 TYR453 LEU455 PHE490 LEU492 LYS493 SER494 TYR495 SER496	2	6
ZINC85546719	−9.4	HIS505 TYR501 SER496 TYR495 SER494 LYS493 PHE490 ILE472 ALA484	3	3
ZINC19764220	−8.9	ARG403 ASP405 GLU406 ARG408 GLN409 SER496 TYR495	1	1
ZINC85543430	−8.8	ARG403 LEU455 TYR453 LEU452 TYR449 SER496 TYR495 SER494 LYS493 LEU492 PHE490 ALA484	2	6

**FIGURE 2:**
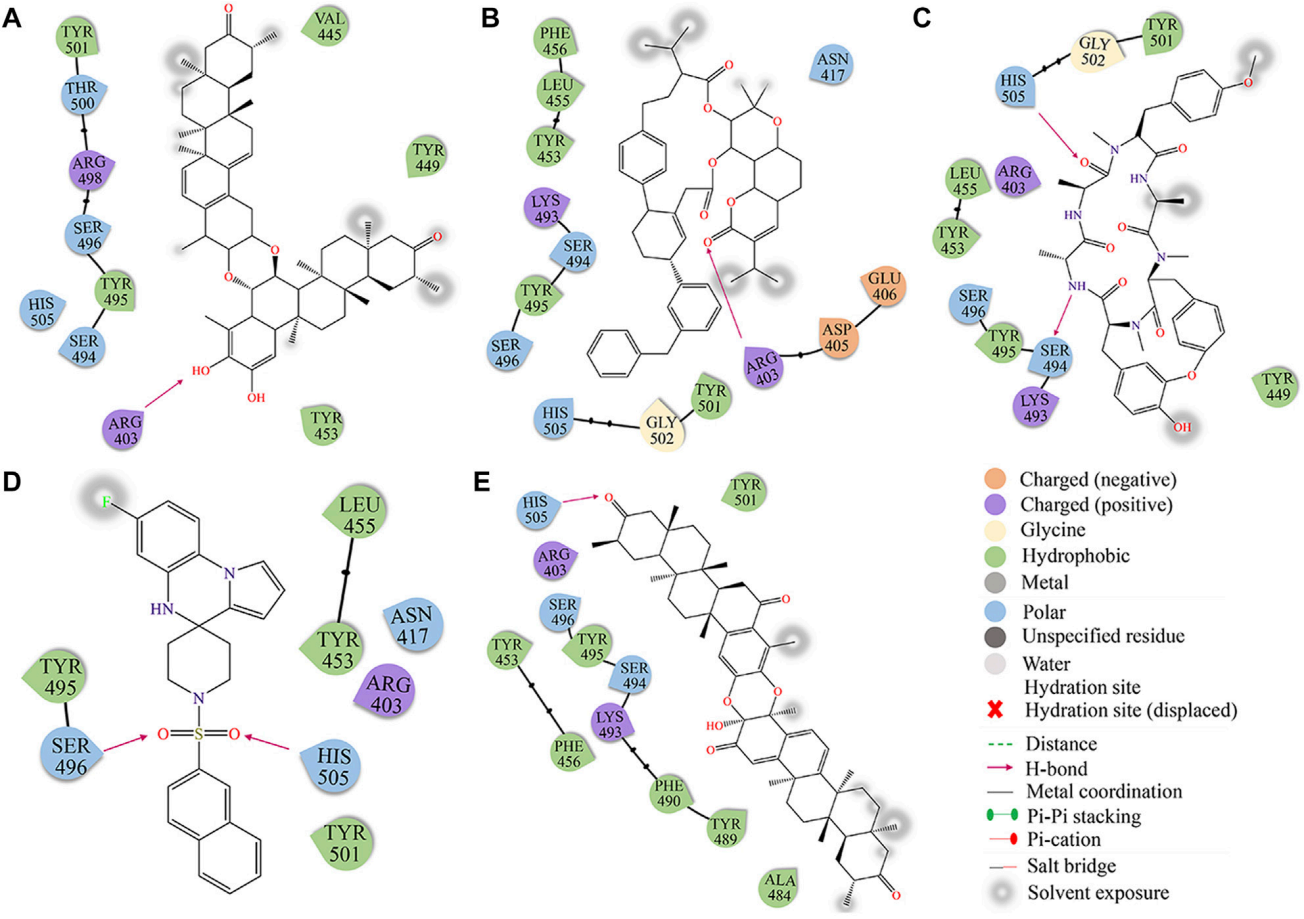
The docking diagram of 2D interaction of RBD protein and TOP5 compound. **(A)** ZINC95919448; **(B)** ZINC85531210; **(C)** ZINC95610651; **(D)** ZINC000035399302; **(E)** ZINC95910594.

The binding mechanism of the top 5 compounds was also analyzed. Interactions of candidate molecules with the RBD protein are presented in Figure 2. ZINC95919448, which had the highest binding affinity (−10.7 kcal/mol), was tightly bound to RBD by interacting with 11 residues. It interacted with ARG403 *via* H-bonds. It also formed hydrophobic interactions with VAL445, TYR449, TYR453, TYR495, and TYR501. ZINC85531210 interacted with ARG403 *via* hydrogen bonds and also interacted with VAL453, TYR455, TYR456, TYR495 and TYR501 *via* hydrophobic interactions. Upon close inspection, residues TYR453, TYR495 and TYR501 were found to be important amino acid residues that form hydrophobic interactions with small molecules, which also exists in ZINC95610651, ZINC000035399302, and ZINC95910594. Additionally, residues SER494 and HIS505 interacted with the ZINC95610651 hydroxy oxygen and amino hydrogen, respectively, through hydrogen bonds. Residues SER496 and HIS505 formed H-bonds with ZINC000035399302 hydroxy oxygen. ZINC95910594 formed hydrogen bonds with HIS505 to enhance the binding affinity to RBD. Candidate compounds (ZINC95919448, ZINC85531210, ZINC95610651, and ZINC95910594) were obtained from the TCMNP database. ZINC000035399302 was obtained from the Alinda natural products database. The close interaction between hot residues and the ACE2 protein increases the probability of the Omicron VOC infecting the human body significantly. The top 5 compounds have strong interactions with hotpot residues, which may prevent Omicron RBD from recognizing ACE2. Notably, their large molecular weight results in a high degree of spatial matching with PPI interfaces with large, flat features (Supplementary Table S1). ZINC000035399302, also known as Deoxybouvardin (RA-V), is a cyclopeptide of the Rubiaceae type that was isolated from *Bouvardia ternifolia* in 1977 (Jolad et al., 1977). Deoxybouvardin and other rubiaceae cyclic peptides are bicyclic cyclic hexapeptides with strong antitumor activity. RA-V is a specific inhibitor of Yes-associated protein (YAP) and transcriptional coactivator with PDZ-binding motif (TAZ) (Ji et al., 2018). RA-V inhibits YAP activation-induced development of liver tumors and is a potential anti-cancer drug. Interestingly, RA-V was found to have anti-inflammatory effects on neutrophil recruitment and edema in carrageenan-induced mouse foot inflammation models (Rupachandra et al., 2020). Moreover, RA-V can also inhibit oxidative stress and expressions of cox as well as TNF-α in inflammatory tissues. RA-V is a potential anti-inflammatory agent. Studies on the other four candidate compounds are not conclusive.

### 3.2 Binding free energy analysis based on MD simulations

The 10 compounds obtained from the previous screening were subjected to final screening *via* Molecular mechanics/Poisson-Boltzmann surface area calculations. The binding free energies were further estimated, including four energy sub-items (ΔEvdW, ΔEele, ΔGpolar, and ΔGnon-polar) based on stable Gromacs trajectories.

The calculation of the contribution of entropy to the binding free energy is a challenging project. Most of approaches that can be used to estimate the entropy of a molecule are time-consuming, and the magnitude of the standard error value is high compared to other contributions. On the other hand, multiple studies have shown that the contribution of net entropy is usually small, and the correction for the change in the free energy of the system configuration leads to only a small improvement in the correlation with the experiment (Brown and Muchmore, 2009; Rastelli et al., 2010; Valdés-Tresanco et al., 2021). When the contribution of entropy is ignored, the value obtained is the effective free energy, which is usually sufficient for comparing relative binding free energies of related ligands, for example to compare different ligands binding to the same receptor protein (Wang et al., 2019; Case et al., 2021).

**FIGURE 3:**
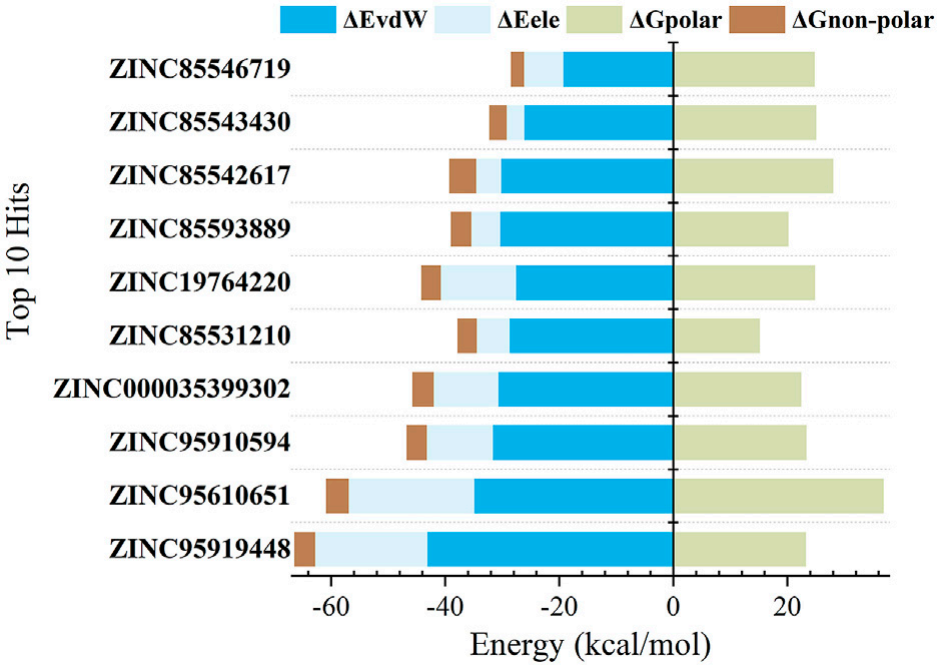
Binding free energy components (kcal/mol) for the binding of top 10 compounds to RBD protein.

The findings are shown in Figure 3 and Table 2. For all candidate molecules, van der Waals and electrostatic interactions were the main contributors of binding energy and electrostatic interactions were responsible for differences in final binding free energy. The weaker the electrostatic effects, the higher the value of the binding free energy (such as ZINC85542617, ZINC85546719, ZINC85543430, ZINC19764220, ZINC85593889). This also leads to a discrepancy between their binding energy and that of other compounds.

**TABLE 2:** Binding free energy details of top10 compounds.

Title	ΔG_VDW_ ^a^	ΔG_Et_ ^b^	ΔG_polar_ ^c^	ΔG_non-polar_ ^d^	ΔG_binding_ ^e^
ZINC95919448	−43.11	−19.67	23.2387	−3.6306	−43.1719
ZINC95610651	−34.8846	−22.0072	36.9099	−3.9896	−23.9715
ZINC95910594	−31.59	−11.6	23.3796	−3.5507	−23.3611
ZINC000035399302	−30.664	−11.3238	22.4537	−3.7545	−23.2886
ZINC85531210	−28.6763	−5.7732	15.1813	−3.3624	−22.6306
ZINC19764220	−27.5309	−13.2513	24.8741	−3.3931	−19.3012
ZINC85593889	−30.3344	−5.08	20.2055	−3.5846	−18.7933
ZINC85542617	−30.1644	−4.4048	28.0515	−4.71	−11.2324
ZINC85543430	−26.0931	−3.0875	25.0464	−3.0579	−7.1921
ZINC85546719	−19.2832	−6.8769	24.8313	−2.2972	−3.626

^a^ van der Waals energy.

^b^ Electrostatic energy.

^c^ Polar-solvation energy.

^d^ Non-polar solvation energy.

^e^ ΔG_binding_ = ΔG_VDW +_ΔG_Et +_ΔG_polar +_ΔG_non-polar_.

Binding free energy rankings of candidate molecules showed striking agreement with docking results. ZINC95919448, ZINC85531210, ZINC95610651, ZINC000035399302, and ZINC95910594 were the top 5 molecules with the highest binding free energy. ZINC95919448 was not only the best performing candidate molecule in docking but also had the lowest binding free energy in MD simulations. The values of its van der Waals interactions (−43.11 kcal/mol) and its binding free energy (−43.18 kcal/mol) were much higher than those of other compounds. ZINC95919448 was the most potential PPI inhibitor. Interestingly, the chemical structures of ZINC95910594 and ZINC000035399302 have very low similarities (Figure 2), but their binding free energy values and each of its terms are almost the same. ZINC85531210 and ZINC95610651 also showed strong binding abilities to the RBD protein (−22.63 kcal/mol, −23.97 kcal/mol). It implies that they have a comparable degree of spatial and energy matching.

**FIGURE 4:**
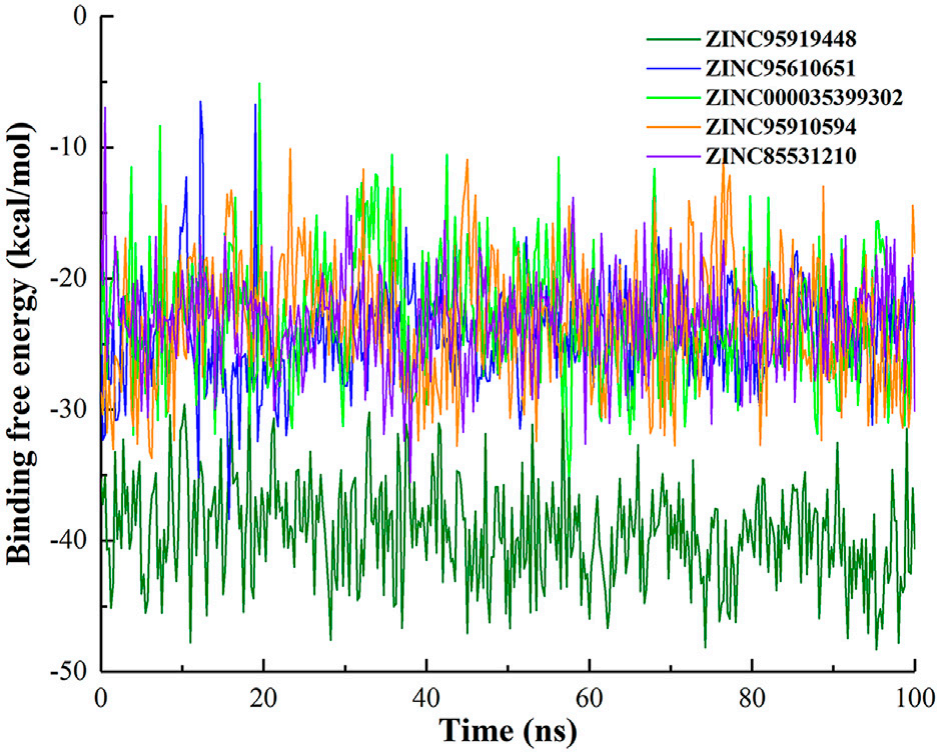
Binding free energy (∆Gbind) over time of top 5 compounds.

To explore the impact of simulation time on binding free energy, binding free energy fluctuations of top10 compounds during the simulation were calculated (Figure 4 and Supplementary Figure S2). The binding free energy of ZINC95919448 with protein remained in dynamic equilibrium during the simulation. It shows that it has a continuous and stable interaction with the protein during the simulation. In the simulation process, most compounds fluctuate greatly in the early stage and maintain the equilibrium state in the late stage (ZINC95610651, ZINC000035399302, ZINC95910594, ZINC85531210, ZINC85593889, ZINC85542617 and ZINC85546719). This is due to the optimization of the initial conformation of the docking in the early simulation, and when the binding conformation reached the lowest energy state, the protein and ligand maintained a stable interaction. Compounds ZINC19764220 and ZINC85543430 showed the largest fluctuation in binding free energy during the simulation, indicating that they did not have stable interaction with proteins, and that they were not ideal binders.

The combined docking results showed that ZINC95919448, ZINC85531210, ZINC95610651, ZINC000035399302, and ZINC95910594 constitute compounds that bind well to RBD proteins. They were then subjected to the MD analysis and prediction of molecular properties.

### 3.3 Molecular dynamics simulations for interaction analyses

**FIGURE 5:**
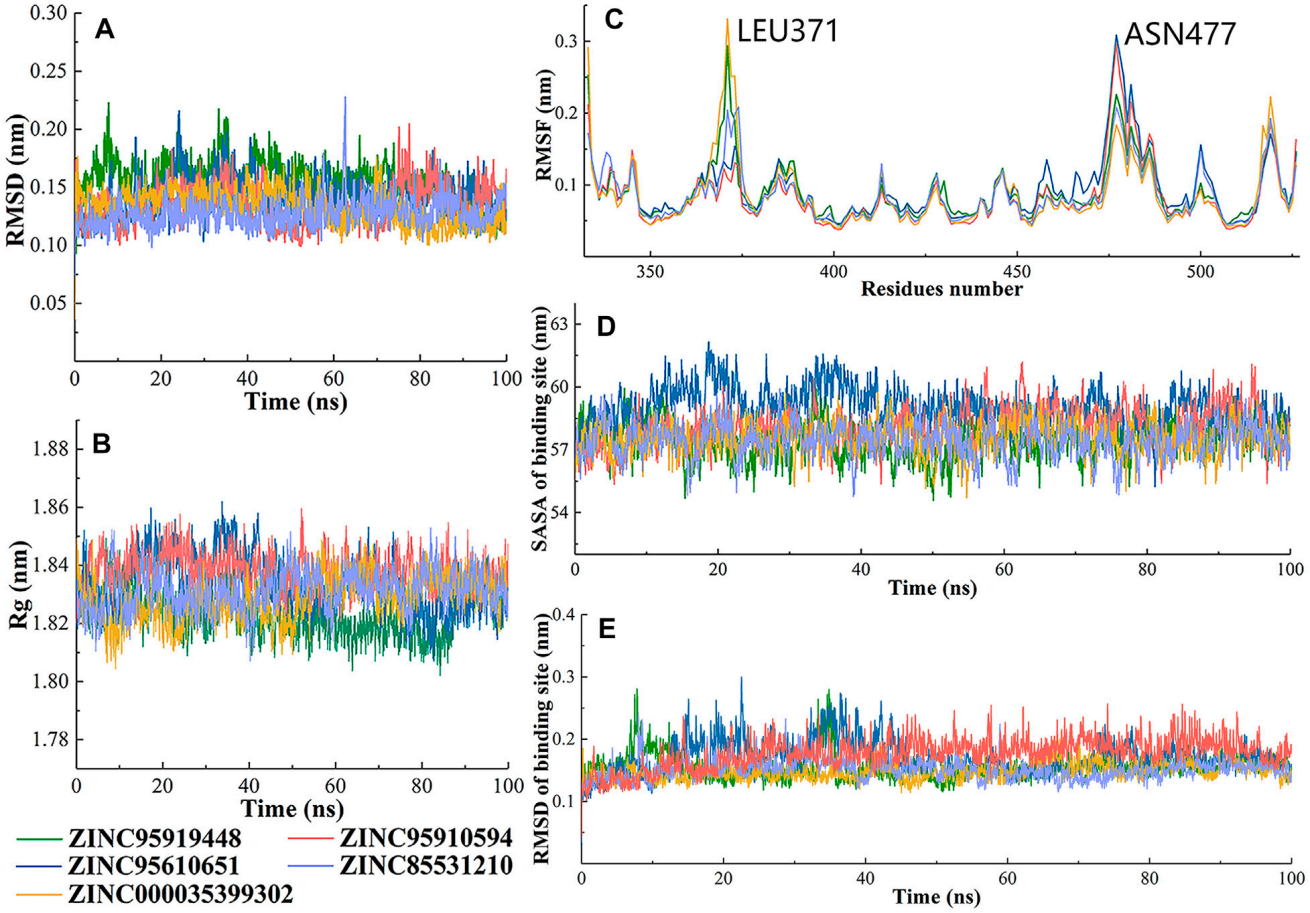
Changes in the RBD structures (5 candidate compounds) and its dynamics with respect to time. **(A)** The RMSD was calculated throughout the MD trajectory simulation time of 100 ns using backbone atoms; **(B)** Radius of gyration of simulated systems; **(C)** The RMSF values of simulated systems were plotted using C-alpha atoms; **(D)** Solvent accessible surface area (SASA) and **(E)** RMSD of binding sites of RBD protein.

The dynamic characteristics of the top5 compounds were calculated during the 100-ns simulation (Figure 5). The root mean square deviations (RMSD) of 5 complexes were analyzed using backbone atoms (Figure 5A). During MD simulation, the RMSD of the five systems fluctuated between 1 and 2 Å. The ZINC95919448 system exhibited slight deviations during 90–100 ns (∼0.3 Å). ZINC85531210 was the system with the smallest fluctuation among the five systems, and the average value of its RMSD was 1.27 Å. The five systems also showed a constant RMSD after 20 ns, implying that they were stable over the entire simulation time and the resulting data was of high confidence. The radius of gyration (Rg) for each frame against the simulated time also showed that the RBD protein that bound with all proposed small molecules attained compactness and rigidity (Figure 5B). The range of Rg values of RBD protein that bound with proposed compounds ZINC95919448, ZINC85531210, ZINC95610651, ZINC000035399302, and ZINC95910594 were 17.9–18.6 Å, 18.0–18.7 Å, 18.0–18.7 Å, 17.9–18.5 Å and 18.0–18.6 Å, respectively. The relatively consistent Rg values also indicated stable folding properties of the protein during the entire MD simulation period.

Fluctuations of protein carbon alpha (Cα) atoms and effects of five candidate molecules binding in the RBD protein were analyzed by root mean square fluctuations plot (Figure 5C). It is reasonable for terminal residues to have high RMSF values. Similar patterns of changes in RMSF values of RBD residues that bound to ZINC95919448 and ZINC000035399302, ZINC85531210 and ZINC95610651 were identified. LEU371 showed the highest RMSF value for ZINC95919448 and ZINC000035399302 systems. LEU371, in a flexible loop and far from the PPI interface, did not affect the results. ASN477 was the most active residue for ZINC95919448 and ZINC000035399302 systems. Although ASN477 is one of the important residues at the RBD-ACE2 PPI interface, it is located at the edge of the RBD protein and did not interact with any of the proposed compounds. Furthermore, other residues (480–505) on the binding surface showed natural fluctuations (1–1.5 Å), indicating that the residues are flexible enough to interact with the ligand.

The RMSD and solvent accessible surface area (SASA) of RBD PPI residues were analyzed (Figures 5D, E). The PPI-RBD RMSD of ZINC000035399302 and ZINC95910594 systems were highly stable which signifies that the residues had the least conformational changes. The convergence of simulation revealed that there were some deviations in RMSD of ZINC95919448, ZINC85531210, and ZINC95610651 systems. The PPI-RBD SASA showed that the ZINC95610651 system was highly exposed to the solvent compared with other complexes (Figure 5E).

**FIGURE 6:**
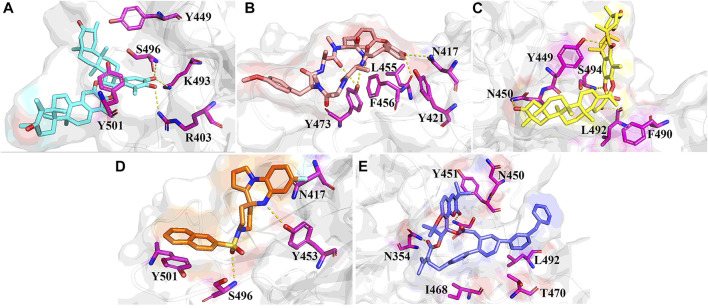
Stable binding conformations between the five candidate compounds and the RBD protein after 100ns MD simulation. **(A)** ZINC95919448; **(B)** ZINC85531210; **(C)** ZINC95610651; **(D)** ZINC000035399302; **(E)** ZINC95910594.

The stable RBD-ligand interaction mode during MD simulation is shown in Figure 6. ZINC95919448 formed hydrogen bonds with three residues and mainly maintained hydrophobic interactions with TYR449 and TYR501 (Figure 6A). A hydrogen bond was formed between the hydroxyl oxygen on the six-membered ring of ZINC95919448 and ARG403, consistent with the docking mode. Each of LYU493and SER496 formed a hydrogen bond with the hydroxyl oxygen, which is important for PPI inhibitions. ZINC95610651 formed a new binding conformation with the RBD protein distinct from docking (Figure 6B). ZINC95610651 formed two hydrogen bonds with TYR473 *via* a hydrogen bond donor and acceptor, respectively. In addition, it formed hydrogen bonds with ASN417 and LEU455. ZINC95910594 formed a close interaction with the PPI interface residues of RBD protein (Figure 6C). TYR449, ASN450, LEU492 and SER494 were involved in the formation of hydrogen bonds. ZINC000035399302 maintained the hydrogen bond with SER496 in the docked conformation and added a hydrogen bond with TYR453, which are necessary for PPI inhibition (Figure 6D). Due to the hydrophobic scaffold of ZINC85531210, it mainly formed hydrophobic interactions with RBD protein, including ASN450, TYR451, ILE468, THR470, and LEU492 among others (Figure 6E).

### 3.4 Binding energy decomposition analysis

**FIGURE 7:**
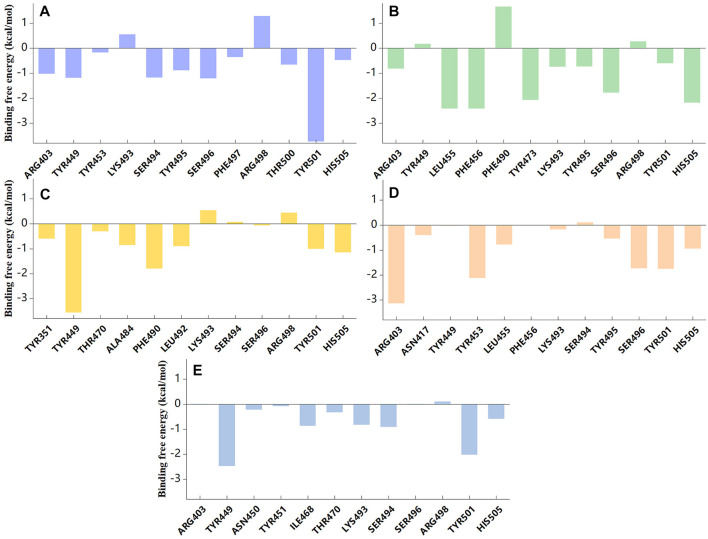
Binding free energy contribution of individual residues of RBD protein during 100ns MD simulation. **(A)** ZINC95919448; **(B)** ZINC85531210; **(C)** ZINC95610651; **(D)** ZINC000035399302; **(E)** ZINC95910594.

As expected, the residues mentioned above that formed interactions with candidate compounds were also found to be significant contributors to the binding (Figure 7, Supplementary Tables S2–S6). Residues TYR501 and HIS505 stabilized small molecules through van der Waals interactions in five systems. ARG403 contributed −1.02 kcal/mol to the binding energy, further demonstrating its important role in ZINC95919448 binding. Moreover, the binding contributions of TYR449, SER494, SER496, and ARG498 to ZINC95919448 and RBD protein were also non-negligible. LEU455, PHE456, TYR473, SER496 played important roles in ZINC95610651 binding to RBD, consistent with findings from the interaction analysis. For ZINC95910594, the contribution of TYR449 was very significant (−3.55 kcal/mol), which may be due to hydrophobic interaction and hydrogen bonding. Residues ARG403, TYR453, and SER496 contributed to the binding of ZINC000035399302 to RBD. For ZINC85531210, TYR449, LYS493, and SER494 had a significant destabilizing effect. Therefore, hotspot residues and other recognition residues were widely involved in the binding of candidate small molecules. These are consistent with the analysis results of stable binding conformation.

From the above interaction analyses and binding energy decomposition analysis, we concluded that (1) more stable and reasonable conformation of the interaction were obtained in MD simulation, suggesting that MD simulations play a role in the refinement of the docking results, (2) the top 5 compounds are the efficient RBD protein binding agents, and (3) all these compounds have stable interactions with the unique mutant residues of Omicron VOC, so they might prevent Omicron from recognizing ACE2.

### 3.5 ADMET analysis

The pharmacokinetic and side effects for the five proposed compounds are presented in Table 3. The blood-brain barrier (BBB) prevents dangerous substances from entering the brain. However, the BBB can block some drugs from entering the brain and the central nervous system. ZINC95919448, ZINC95610651, and ZINC85531210 could not penetrate the BBB, while ZINC85531210 was shown to penetrate the BBB. Apart from ZINC95910594, all other compounds were absorbed through the gastrointestinal tract. P-glycoprotein (P-gp) is an important protein of the cell membrane that pumps many foreign substances out of cells. ZINC95610651 was not shown to be a substrate for P-gp while ZINC000035399302 was a P-gp inhibitor. All candidate molecules showed very low inhibitions of cytochrome P450 enzymes (CYP). ZINC000035399302 had a good performance with regards to Caco-2 cell permeability exhibiting the permeability of molecules into the large intestines. ZINC95919448 and ZINC95910594 do not cause severe drug-induced liver injury.

**TABLE 3:** Predicted toxicity of screened compounds with suitable affinity.

Title	BBB	HIA	Pgp substrate	Pgp inhibitor	CYP1A2	CYP2C9	CYP2C19	CYP2D6	CYP3A4	Caco-2 permeability	Dili	hERG blockers
ZINC95919448	NO	YES	YES	NO	NO	NO	NO	YES	YES	NO	NO	YES
ZINC95610651	NO	YES	NO	NO	NO	NO	NO	NO	YES	NO	YES	NO
ZINC95910594	YES	NO	YES	NO	NO	NO	NO	NO	YES	NO	NO	NO
ZINC000035399302	YES	YES	YES	YES	YES	YES	YES	NO	YES	YES	YES	YES
ZINC85531210	NO	YES	YES	NO	NO	YES	YES	NO	YES	NO	YES	NO

### 3.6 Molecular electrostatic potential (MEP)

**FIGURE 8:**
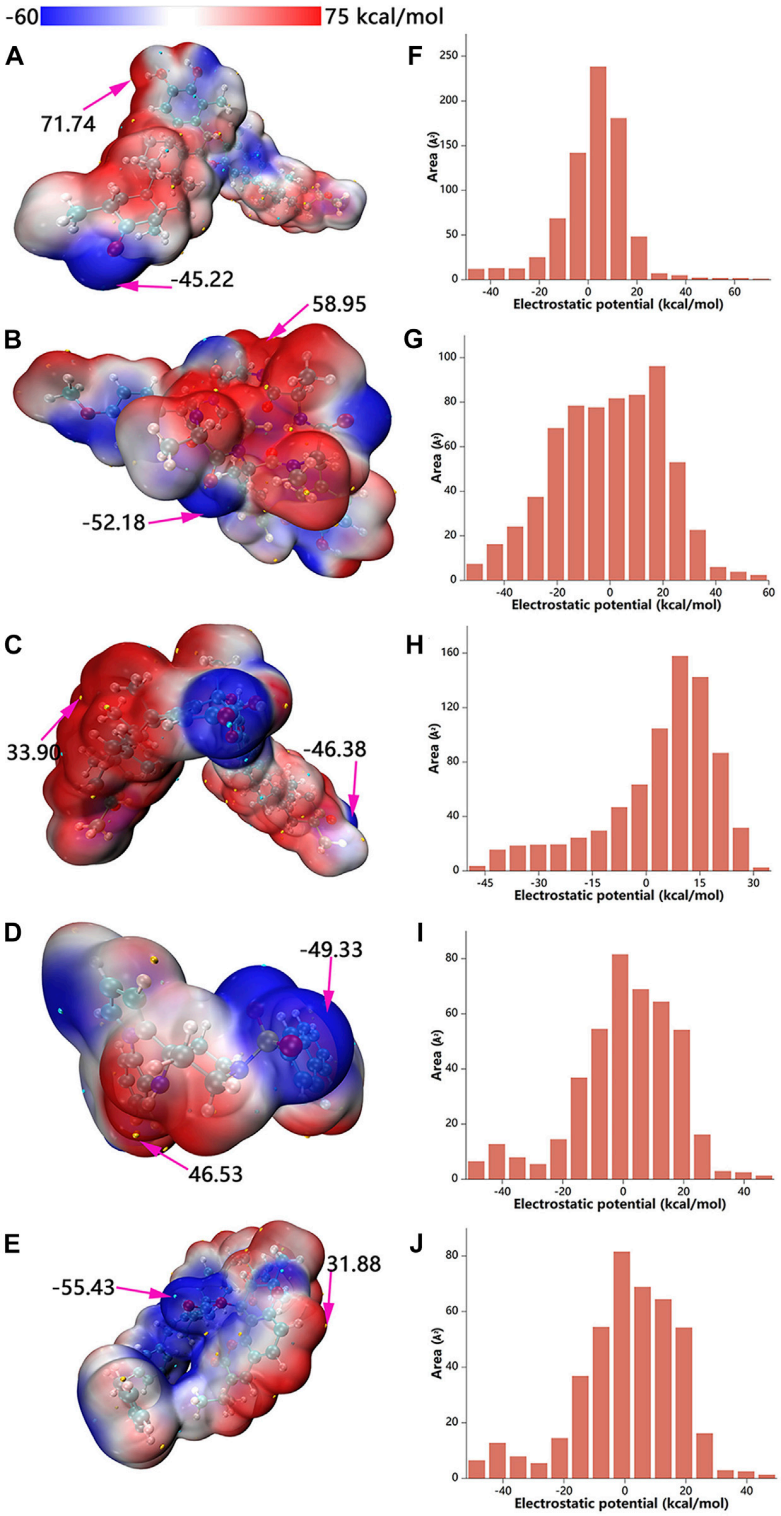
Electrostatic potential mapped molecular vdW surface **(A–E)** and surface area in each ESP range **(F–J)**. A, F: ZINC95919448; B, G: ZINC85531210; C, H: ZINC95610651; D, I: ZINC000035399302; E, J: ZINC95910594.

The maxima and minima of candidate molecular ESPs were localized on the vdW surface by quantitative molecular surface analysis (Figures 8A–E). In addition, the distributions of different ESP intervals on the vdW surface were calculated (Manzetti and Lu, 2013; Lu and Manzetti, 2014) and plotted (Figures 8F–J). With regards to color scale in the right side of the figure, negative values (blue) display the electron-rich negatively charged part of the molecule, while positive values (red) indicate opposite characteristics. The ubiquitous presence of red and blue in the ESP map demonstrates the polar character of the molecule, with the blue part of the map more likely to interact with the positively charged residues or the red part with the negatively charged residues. The median value of ESP (white) represents the weakly polar hydrophobic part of the molecule. As shown in from the labelled text in Figures 8A–E, the ESP of the vdW surface of ZINC95919448 ranges from −45.22–71.74 kcal/mol, which is a wider range relative to the other four molecules, indicating that ZINC95919448 might form strong electrostatic interactions with amino acid residues. ZINC95919448 and ZINC95910594 tended to act as a Lewis acid to bound Lewis base species. ZINC000035399302 and ZINC85531210 tended to act more as a Lewis base to dock to the Lewis acid species. ZINC95910594 had no obvious tendency. This was based on comparisons of the magnitude of ESP at the maximum and minimum on the vdW surface as well as on comparisons of the surface area of positive and negative ESPs. The electrostatic potential is one of the fundamentals of electrostatic interaction. Because electrostatic interaction is the primary long-distance interaction between molecules. Electrostatic potential plays a very unique role in the interaction of inter-molecules and protein, the reaction site of molecules in metabolism, and molecular recognition.

### 3.7 Molecular properties analysis based on density functional reaction theory

The following descriptors were obtained: chemical hardness (η), chemical softness (S), band gap (GAPE), electron affinity (EA), ionization potential (IP), electrophilicity index (ω), nucleophilicity index (N), electronic potential (μ), and electronegativity (χ) (Table 4). Based on Koopman’s theorem (LIU, 2016), opposite numbers of the values of HOMOs and LUMOs correspond to the electron affinity (EA) and ionization potential (IP), respectively. Bases on the Pearson’s Hard/Soft Acid-Base (HSAB) principle, Chemicals can be described as hard or soft acids or bases (Parr and Pearson, 1983). Hard species are small in volume, difficult to polarize, highly charged, and have a large HOMO-LUMO gap, while soft species are the opposite. ZINC95919448 has lower ionization potential (IP = 5.16 eV), electron affinity (EA = −0.46 eV) and chemical hardness (η = 6.9 eV) values, which support its reactivity. Nucleophilicity is an important parameter that can reflect the reactivity of compounds. ZINC95919448 has strong nucleophilicity (N = 3.96 eV), while ZINC85531210 has soft nucleophilicity (N = 3.23 eV). Quantum chemical descriptors are of great significance for evaluating the molecular properties and guiding experiments.

**TABLE 4:** The Quantum chemical descriptors at B3LYP-D3/6-311G** level.

Compounds	HOMO	LUMO	η	S	GAPE	EA	IP	ω	N	μ	χ
ZINC95919448	−5.16	0.46	6.9	0.14	5.62	−0.46	5.16	0.65	3.96	−2.99	2.99
ZINC95610651	−5.48	0.77	7.48	0.13	6.25	−0.77	5.48	0.59	3.64	−2.97	2.97
ZINC95910594	−5.82	−1.15	5.85	0.17	4.67	1.15	5.82	1.42	3.3	−4.07	4.07
ZINC000035399302	−5.57	0.02	7.07	0.14	5.59	−0.02	5.57	0.87	3.55	−3.51	3.51
ZINC85531210	−5.89	−0.27	6.7	0.15	5.62	0.27	5.89	0.98	3.23	−3.62	3.62

## 4 Conclusion

The COVID-19 disease has become a global burden affecting human health. Advent of the Omicron VOC has increased the number of deaths and infections. Computer-aided and structure-based design of drugs of protein targets have incredibly accelerated drug discovery. The PPI interface inhibitors evaluated through this method bind well to known target proteins.

We screened for high potential inhibitors (ZINC95919448, ZINC85531210, ZINC95610651, ZINC000035399302, and ZINC95910594) against the Omicron VOC RBD. First, the five top-performing compounds were screened from 92, 699 compounds *via* double docking, molecular dynamics simulations and binding free energy analysis. Secondly, protein ligand-interaction analysis, MD simulation trajectory analysis, and binding free energy decomposition were combined to assess their mechanism as inhibitors at the molecular level, and predicted their pharmacokinetics as well as other related data. Finally, molecular electrostatic potentials and other quantitative computational descriptors were calculated to show their chemical properties at the electronic level. The five candidate compounds (ZINC95919448, ZINC85531210, ZINC95610651, ZINC000035399302, and ZINC95910594) were shown to interact with hotspot residues on the Omicron RBD protein *via* strong electrostatic interactions, including hydrogen bonding and hydrophobicity, thereby effectively interfering with Omicron VOC RBD protein recognition of human ACE2 receptor protein, preventing the virus spread. *In vitro* and *in vivo* studies should be performed to assess the potential of these compounds in inhibiting the Omicron VOC infections.

## Data Availability

The original contributions presented in the study are included in the article/Supplementary Material; further inquiries can be directed to the corresponding authors.
