# Supplementary material for: Identification of potential inhibitors of omicron variant of SARS-Cov-2 RBD based virtual screening, MD simulation, and DFT
Source: Front Chem. 2022 Dec 8;10:1063374. doi: 10.3389/fchem.2022.1063374 (PMC9772825; doi:10.3389/fchem.2022.1063374)
Supplement: Supplementary file 1 [file DataSheet2.docx]

Supplementary Material

# Supplementary Figures


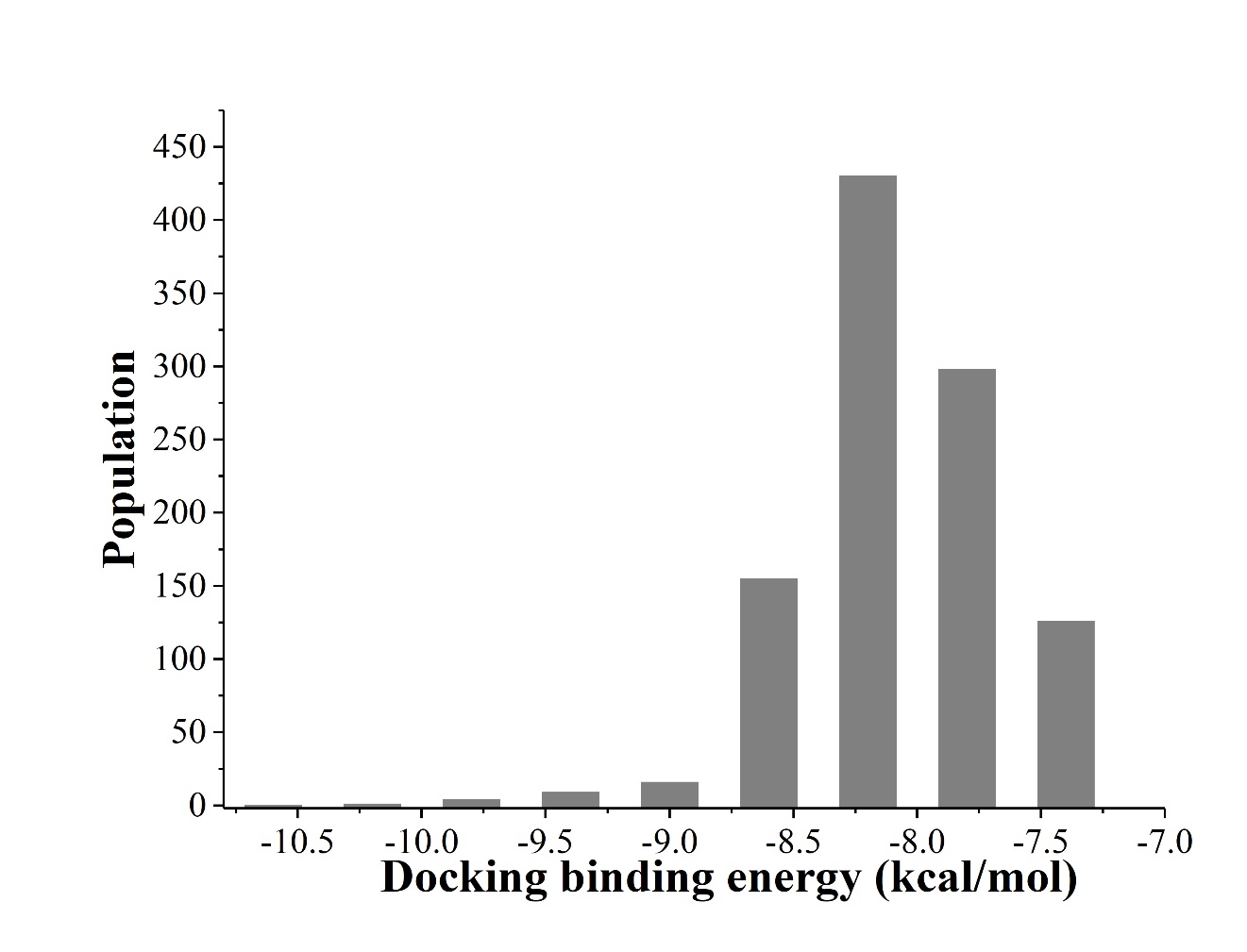


**Figure S1** Results of virtual screening using AutoDock Vina of the small molecule compound database against SARS-CoV-2 Omicron RBD protein. Bars represent numbers of compounds with predicted free energies of binding in the indicated 0.4 kcal/mol bins.

**Figure S2** Binding free energy (∆Gbind) over time of top 6-10 compounds.
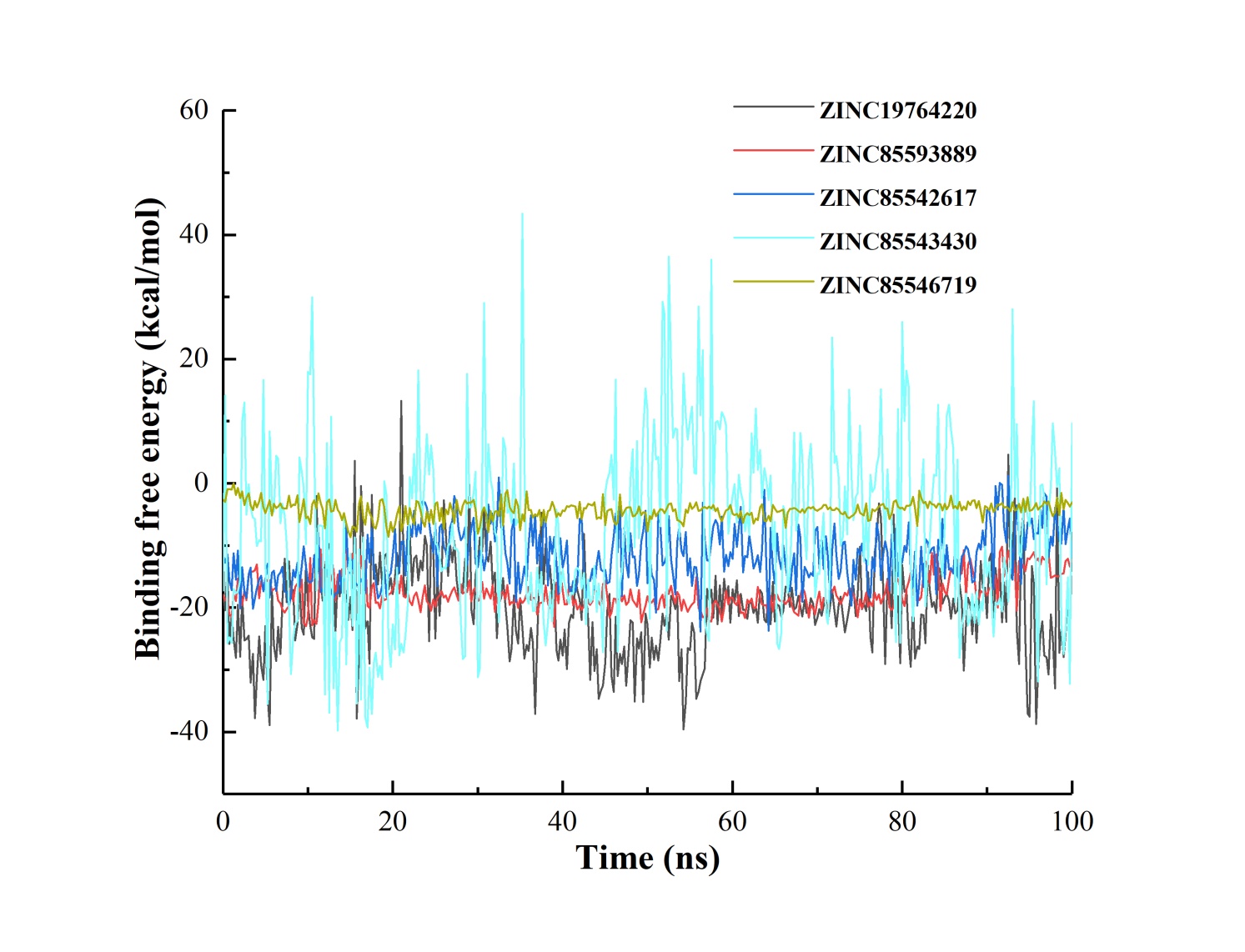


# Supplementary Tables

**Table S1** Docking results and details of top50 compounds

| No. | ZINC ID | Molecular formula | Database | ΔG | NRR | Interactions (H-bond) | MW |
| --- | --- | --- | --- | --- | --- | --- | --- |
| 1 | ZINC95919448 | C56H72O6 | TCMNP | -10.7 | 8 | ARG403 | 841.2 |
| 2 | ZINC85531210 | C51H52O7 | TCMNP | -10 | 9 | ARG403 | 777 |
| 3 | ZINC95610651 | C40H48N6O9 | TCMNP | -9.9 | 9 | SER494 HIS505 | 756.9 |
| 4 | ZINC000035399302 | C25H22FN3O2S | Alinda | -9.8 | 5 | SER496 HIS505 | 447.5 |
| 5 | ZINC95910594 | C56H72O7 | TCMNP | -9.7 | 8 | HIS505 | 857.2 |
| 6 | ZINC85542617 | C51H67NO3 | TCMNP | -9.6 | 6 | Y453 | 742.1 |
| 7 | ZINC85593889 | C44H45N6O7 | TCMNP | -9.6 | 6 | K493 H505 | 769.9 |
| 8 | ZINC85546719 | C48H72N6O4 | TCMNP | -9.4 | 3 | — | 797.1 |
| 9 | ZINC19764220 | C26H24N4O3 | Asinex | -8.9 | 1 | HIS505 | 440.5 |
| 10 | ZINC85543430 | C49H65NOS2 | TCMNP | -8.8 | 6 | R403 | 748 |
| 11 | ZINC08221305 | C42H58N2O | TCMNP | -8.8 | 5 | — | 607 |
| 12 | ZINC85570057 | C42H58O9 | TCMNP | -8.8 | 5 | — | 707 |
| 13 | ZINC85592456 | C46H43N2O10 | TCMNP | -8.8 | 2 | — | 783.8 |
| 14 | ZINC85568707 | C33H30N2O10 | TCMNP | -8.8 | 2 | R403 | 614.6 |
| 15 | ZINC85542728 | C47H65NO3S2 | TCMNP | -8.8 | 6 | — | 756.2 |
| 16 | ZINC95918800 | C42H42N4O3 | TCMNP | -8.8 | 4 | — | 650.8 |
| 17 | ZINC85568720 | C33H34N4O10 | TCMNP | -8.8 | 1 | — | 646.7 |
| 18 | ZINC85592463 | C44H41N2O10 | TCMNP | -8.8 | 5 | R403 | 757.8 |
| 19 | ZINC85542671 | C48H66N2O3 | TCMNP | -8.8 | 3 | — | 719.1 |
| 20 | ZINC245339471 | C22H22F3NO8 | Alinda | -8.7 | 3 | — | 485.4 |
| 21 | ZINC33353750 | C36H36N6 | Alinda | -8.7 | 4 | — | 552.7 |
| 22 | ZINC85592453 | ZINC85592453 | TCMNP | -8.7 | 4 | R403 N417 | 773.8 |
| 23 | ZINC169289767 | C34H28N6O14S4 | ADMJ | -8.7 | 5 | R403 | 872.9 |
| 24 | ZINC85542926 | C41H61NO3 | TCMNP | -8.7 | 2 | — | 616 |
| 25 | ZINC95918631 | C26H30O10 | TCMNP | -8.7 | 4 | R403 | 502.5 |
| 26 | ZINC85593850 | C42H39N6O7S2 | TCMNP | -8.7 | 4 | H505 | 803.9 |
| 27 | ZINC85568704 | C31H28N4O10 | TCMNP | -8.7 | 1 | R403 | 616.6 |
| 28 | ZINC85530296 | C35H38O9 | TCMNP | -8.7 | 6 | R403 | 602.7 |
| 29 | ZINC85593529 | C30H27NO8 | TCMNP | -8.7 | 3 | K493 H505 | 529.5 |
| 30 | ZINC85593830 | ZINC85593830 | TCMNP | -8.7 | 3 | — | 812.9 |
| 31 | ZINC85592449 | C47H45N2O11 | TCMNP | -8.7 | 3 | R403 R498 | 813.9 |
| 32 | ZINC85541924 | C28H34O6 | TCMNP | -8.7 | 3 | — | 466.6 |
| 33 | ZINC85542793 | C45H67NO3 | TCMNP | -8.7 | 5 | — | 670 |
| 34 | ZINC751853 | C25H23NO4S | Alinda | -8.6 | 4 | — | 433.5 |
| 35 | ZINC70691824 | C27H40O4 | TCMNP | -8.6 | 3 | — | 428.6 |
| 36 | ZINC64848214 | C25H29N3O2S | Alinda | -8.6 | 4 | — | 435.6 |
| 37 | ZINC100574268 | C30H30N2O3 | Alinda | -8.6 | 3 | H505 | 466.6 |
| 38 | ZINC85595889 | C50H55N3O8 | TCMNP | -8.6 | 4 | — | 826 |
| 39 | ZINC85542935 | C43H65NO3 | TCMNP | -8.6 | 6 | — | 644 |
| 40 | ZINC85568676 | C47H50N2O10 | TCMNP | -8.6 | 4 | — | 803 |
| 41 | ZINC85568245 | C53H57N3O7 | TCMNP | -8.6 | 4 | R403 | 848.1 |
| 42 | ZINC164528615 | C38H46F4N6O9S | ADMJ | -8.6 | 3 | — | 838.9 |
| 43 | ZINC85571170 | C36H42N2O4S2 | TCMNP | -8.6 | 3 | — | 630.9 |
| 44 | ZINC85594934 | C30H30N7O7 | TCMNP | -8.6 | 4 | E406 H505 | 600.6 |
| 45 | ZINC58090636 | C24H19NO5 | Alinda | -8.6 | 1 | — | 401.4 |
| 46 | ZINC2400723 | C26H27N3O | Alinda | -8.6 | 2 | R403 | 397.5 |
| 47 | ZINC239347304 | C31H39NO4 | Alinda | -8.6 | 1 | — | 489.7 |
| 48 | ZINC11691930 | C27H20O5 | Alinda | -8.6 | 3 | — | 424.4 |
| 49 | ZINC85592445 | C46H41N2O11 | TCMNP | -8.6 | 5 | R403 N417 | 797.8 |
| 50 | ZINC85627228 | C45H66N6O6S2 | TCMNP | -8.6 | 4 | V524 | 851.2 |

ΔG: Docking binding energy (kcal/mol); NRR: The number of recognition residues; MW: Molecule weight (g/mol); ADMJ: Approved Drugs in Major Juridications.

**Table S2** Energetic decomposition of the contribution of the binding residues to binding free energy for ZINC95919448 during MD simulation (kcal/mol)

| Residues | ΔG_VDW_ | ΔG_Et_ | ΔG_polar_ | ΔG_apolar_ | ΔG_binding_ |
| --- | --- | --- | --- | --- | --- |
| ARG403 | -1.25 | -0.56 | 0.84 | -0.05 | -1.02 |
| TYR449 | -1.29 | -0.26 | 0.62 | -0.25 | -1.18 |
| TYR453 | -0.18 | -0.03 | 0.07 | -0.02 | -0.16 |
| LYS493 | -0.72 | -0.48 | 1.95 | -0.19 | 0.56 |
| SER494 | -0.06 | -2.57 | 1.51 | -0.05 | -1.17 |
| TYR495 | -0.86 | -0.44 | 0.46 | -0.04 | -0.88 |
| SER496 | -2.21 | -0.77 | 2.05 | -0.27 | -1.2 |
| PHE497 | -0.32 | 0.03 | -0.06 | 0 | -0.35 |
| ARG498 | -1.12 | 0.71 | 1.88 | -0.17 | 1.3 |
| THR500 | -1.89 | -0.08 | 1.7 | -0.38 | -0.65 |
| TYR501 | -4.81 | 0.03 | 1.56 | -0.51 | -3.73 |
| HIS505 | -1.38 | -0.26 | 1.45 | -0.27 | -0.46 |

**Table S3** Energetic decomposition of the contribution of the binding residues to binding free energy for ZINC95610651 during MD simulation (kcal/mol)

| Residues | ΔG_VDW_ | ΔG_Et_ | ΔGpolar | ΔGapolar | ΔGbinding |
| --- | --- | --- | --- | --- | --- |
| ARG403 | -0.74 | -0.57 | 0.51 | -0.01 | -0.81 |
| TYR449 | 0.41 | -1.04 | 0.87 | -0.06 | 0.18 |
| LEU455 | -2.01 | -0.68 | 0.31 | -0.03 | -2.41 |
| PHE456 | -1.93 | -0.09 | 0.19 | -0.58 | -2.41 |
| PHE490 | -0.73 | 2.8 | 0.27 | -0.67 | 1.67 |
| TYR473 | -1.44 | -0.98 | 0.98 | -0.62 | -2.06 |
| LYS493 | -0.63 | -0.55 | 0.92 | -0.48 | -0.74 |
| TYR495 | -0.9 | -0.17 | 0.71 | -0.36 | -0.72 |
| SER496 | -1.43 | -1.15 | 1.62 | -0.81 | -1.77 |
| ARG498 | -0.58 | 0.95 | 0.53 | -0.62 | 0.28 |
| TYR501 | -0.66 | -0.47 | 0.83 | -0.29 | -0.59 |
| HIS505 | -1.64 | -0.36 | 0.59 | -0.76 | -2.17 |

**Table S4** Energetic decomposition of the contribution of the binding residues to binding free energy for ZINC95910594 during MD simulation (kcal/mol)

| Residues | ΔG_VDW_ | ΔG_Et_ | ΔGpolar | ΔGapolar | ΔGbinding |
| --- | --- | --- | --- | --- | --- |
| TYR351 | -0.72 | -0.06 | 0.34 | -0.15 | -0.59 |
| TYR449 | -3.62 | -0.35 | 0.98 | -0.56 | -3.55 |
| THR470 | -0.7 | -0.05 | 0.6 | -0.15 | -0.3 |
| ALA484 | -0.03 | -0.02 | 0.04 | -0.84 | -0.85 |
| PHE490 | -1.8 | -0.2 | 0.55 | -0.34 | -1.79 |
| LEU492 | -1.34 | 0.14 | 0.42 | -0.12 | -0.9 |
| LYS493 | -0.59 | -0.12 | 1.31 | -0.06 | 0.54 |
| SER494 | -1.17 | -0.03 | 1.51 | -0.23 | 0.08 |
| SER496 | -0.37 | 0.01 | 0.37 | -0.08 | -0.07 |
| ARG498 | -0.17 | -0.12 | 0.77 | -0.04 | 0.44 |
| TYR501 | -0.96 | -0.36 | 0.62 | -0.31 | -1.01 |
| HIS505 | -1.45 | -0.25 | 0.71 | -0.16 | -1.15 |

**Table S5** Energetic decomposition of the contribution of the binding residues to binding free energy for ZINC000035399302 during MD simulation (kcal/mol)

| Residues | ΔG_VDW_ | ΔG_Et_ | ΔGpolar | ΔGapolar | ΔGbinding |
| --- | --- | --- | --- | --- | --- |
| ARG403 | -2.29 | -1.99 | 1.43 | -0.28 | -3.13 |
| ASN417 | -0.41 | -0.05 | 0.13 | -0.07 | -0.4 |
| TYR449 | -0.09 | -0.01 | 0.09 | -0.01 | -0.02 |
| TYR453 | -1.23 | -1.6 | 0.75 | -0.04 | -2.12 |
| LEU455 | -0.72 | -0.03 | 0.15 | -0.18 | -0.78 |
| PHE456 | -0.08 | 0.03 | 0.05 | -0.01 | -0.01 |
| LYS493 | -0.71 | -1.01 | 1.71 | -0.16 | -0.17 |
| SER494 | -0.31 | 0.31 | 0.13 | -0.02 | 0.11 |
| TYR495 | -1.19 | -0.53 | 1.39 | -0.21 | -0.54 |
| SER496 | -1.4 | -1.6 | 1.48 | -0.21 | -1.73 |
| TYR501 | -2.48 | -0.16 | 1.2 | -0.31 | -1.75 |
| HIS505 | -2.16 | 0.92 | 0.61 | -0.31 | -0.94 |

**Table S6** Energetic decomposition of the contribution of the binding residues to binding free energy for ZINC85531210 during MD simulation (kcal/mol)

| Residues | ΔG_VDW_ | ΔG_Et_ | ΔGpolar | ΔGapolar | ΔGbinding |
| --- | --- | --- | --- | --- | --- |
| ARG403 | -0.01 | 0.29 | -0.27 | 0.01 | 0.02 |
| TYR449 | -2.82 | -0.34 | 1.24 | -0.55 | -2.47 |
| ASN450 | -0.41 | -0.09 | 0.3 | -0.01 | -0.21 |
| TYR451 | -0.29 | -0.01 | 0.25 | -0.02 | -0.07 |
| ILE468 | -1.25 | -0.24 | 0.98 | -0.35 | -0.86 |
| THR470 | -0.52 | -0.12 | 0.37 | -0.05 | -0.32 |
| LYS493 | -0.66 | 0.09 | -0.14 | -0.11 | -0.82 |
| SER494 | -0.78 | -1.7 | 1.72 | -0.14 | -0.9 |
| SER496 | -0.09 | 0.08 | 0.03 | -0.01 | 0.01 |
| ARG498 | -0.08 | 0.23 | -0.03 | -0.01 | 0.11 |
| TYR501 | -1.12 | 0.01 | 0.01 | -0.92 | -2.02 |
| HIS505 | -1.01 | 0.03 | -0.01 | 0.41 | -0.58 |
